# Supplementary material for: Bank liquidity and the risk-taking channel of monetary policy: An empirical study of the banking system in China
Source: PLoS One. 2022 Dec 27;17(12):e0279506. doi: 10.1371/journal.pone.0279506 (PMC9794090; doi:10.1371/journal.pone.0279506)
Supplement: Attachment — Submitted filename: Response to Reviewers.doc [file pone.0279506.s002.doc]

Rebuttal Letter

Dear Prof. Nicolitsas,

We would like to thank PLOS ONE for giving us the opportunity to revise our manuscript.
We have checked the manuscript and revised it according to the comments. We submit here the revised manuscript as well as a list of changes.
If you have any question about this paper, please don’t hesitate to let me know.

Sincerely yours,
Lihuan Zhuang

**Response to Journal Requirements:**

1. We have ensure our manuscript meets PLOS ONE's style requirements.
2. We have removed the funding information from our manuscript and we don’t need to update our Funding Statement in the Funding Statement section of the online submission form.
3. The role of the funders took in the study is stated as follows: "The funders had no role in study design, data collection and analysis, decision to publish, or preparation of the manuscript."

**Response to Reviewer #1:**

We appreciated the constructive criticism and suggestion of Reviewer #1. We addressed all the points raised by the reviewer, as summarized below.

1. The concept of liquidity risk-taking channel of monetary policy has not been used in the banking literature. Khan et al.(2017) and Acharya and Naqvi(2012) just investigate the relationship between liquidity and bank risk without monetary policy, while Borio and Zhu (2012) and Chen et al. (2017) focus on the relationship between monetary policy and bank risk, and bank liquidity is treated as a control variable. According to the referee’s suggestion, we strengthen our gap analysis to further explain how our research relates to and contributes beyond the findings documented in the literature.
2. According to the referee’s advice, we present a graph of the changes in bank liquidity about the Chinese banking sector during the observation period (2003-2018) in the Introduction section.
3. Thanks to the referee’s comment, we strengthen our gap analysis in the Introduction section and Hypothesis development section
4. To verify the robustness of the results, we use Loans to Deposits ratio as alternative measures of liquidity risk to run robustness checks.
5. We improve the analysis of the estimated coefficients of the interaction term and control other bank-specific characteristics and Crisis dummy indicator in the regressions.
6. According to the referee’s suggestion, we further explain the purpose of using the mediation model and extend the analysis of the estimation results.
7. Thanks to the referee’s comment, we further formulate policy implications for bank managers and regulators.

**Response to Reviewer #2:**

We want to thank referee #2 for constructive and insightful criticism and advice. We addressed all the points raised by the reviewer as summarized below.

1. According to the referee’s suggestion, we focus on the transmission mechanism of the risk-taking channel of monetary policy through bank liquidity and extend the analysis related to Hypothesis 2.
2. We strengthen our gap analysis in the Introduction section and Hypothesis development section to thoroughly explain how our research relates to and contributes beyond the findings documented in the literature.
3. According to the referee’s suggestion, we remove MP from the model (2) .
4. Considering the possible endogeneity of Liquidity, we adopt its first lag value in the regressions. However, as the MP variables are treated as exogenous, we haven’t treated MP with a lag related to the dependent variable banks’ Z-score.
5. Thanks to the referee’s comment, we reset the model (3) by including MP, Liquidity, and MP×Liquidity.
6. To be consistent in the tests, we use three variables (i.e. reserve, interbank, M2) to proxy for the monetary policy in each models.
7. According to the referee’s suggestion, we further explain the purpose of using the mediation model and extend the analysis of the estimation results related to Hypothesis 2.
